# The association between polyunsaturated fatty acids and periodontitis: NHANES 2011–2014 and Mendelian randomisation analysis

**DOI:** 10.1186/s12944-024-02159-0

**Published:** 2024-06-04

**Authors:** Tao Li, Huadong Wu, Zhenzhen Fu, Hong Li, Quan Li, Yi Liu, Qiang Zhang

**Affiliations:** Department of Oral and Maxillofacial Surgery, The First Affiliated Hospital, Jiangxi Medical College, Nanchang University, Jiangxi, China

**Keywords:** NHANES, Periodontitis, RCS, Mendelian randomisation analysis, PUFA

## Abstract

**Background:**

We aimed to explore the association and potential causality between polyunsaturated fatty acids concentrations and the risk of periodontal disease.

**Materials and methods:**

Data were collected from the 2011–2014 National Health and Nutrition Examination Survey (NHANES). Weighted logistic regression analysis and restricted cubic spline (RCS) analysis were used to analyse the associations of the concentrations of omega-3 and omega-6 fatty acids and the omega-6/omega-3 fatty acids ratio with the risk of periodontitis. E-value and propensity score matching (PSM) analyses were used for sensitivity analyses. In addition, two-sample Mendelian randomisation (MR) analyses were performed to assess the potential causal impact of the concentrations of those fatty acids on periodontitis risk.

**Results:**

A total of 2462 participants from the NHANES were included. Logistic regression analysis revealed that high omega-3 fatty acids levels were negatively associated with the risk of developing periodontitis (*P* < 0.05), while the omega-6/omega-3 fatty acids ratio was positively associated with the risk of developing periodontitis (*P* < 0.05). There was no significant association between omega-6 concentrations and the risk of periodontitis. The findings mentioned above were confirmed by analysis following a 1:1 PSM. Furthermore, MR examination of the two samples indicated no possible causal link between the risk of periodontitis and the concentrations of omega-3 or omega-6 fatty acids or the ratio of omega-6 to omega-3 fatty acids (*P* > 0.05).

**Conclusion:**

Although omega-3 fatty acids and the omega-6/omega-3 fatty acids ratio were associated with the risk of periodontitis in cross-sectional studies, the MR results did not support a causal relationship between them. Therefore, there is no indication that an increase in the omega-3 fatty acids concentration or a decrease in the omega-6/omega-3 fatty acids ratio may be beneficial for preventing periodontitis.

**Supplementary Information:**

The online version contains supplementary material available at 10.1186/s12944-024-02159-0.

## Introduction

Periodontitis is a chronic inflammatory disease of tooth-supporting tissues comprising the gingiva, cementum, periodontium, and alveolar bone [1]. It is estimated that more than half of the global population is affected by this disease [2]. In 2019, there were an estimated 1.1 billion severe periodontitis cases worldwide [3]. It is anticipated that this number will increase as the population ages. Moreover, it is estimated that severe periodontitis leads to an annual global loss in productivity of 54 billion dollars [4]. Furthermore, periodontitis is strongly associated with various systemic diseases, such as Alzheimer’s disease, adverse pregnancy outcomes, rheumatoid arthritis, cardiovascular disease, and diabetes [5–7]. Consequently, preventing the occurrence of periodontitis is crucial not only to oral health but also to systemic health.

Studies have confirmed that dietary intake is a modifiable factor in periodontal disease [8]. Polyunsaturated fatty acids (PUFA) are essential fatty acids that cannot be synthesized by the human body. According to the different positions of the first double bond, PUFA can be divided into two major categories: omega-3 fatty acids (n-3) and omega-6 fatty acids (n-6) [9]. Among these fatty acids, omega-3 fatty acids intake has been demonstrated to exhibit anti-inflammatory effects and is linked to a decreased risk of developing depression, dementia, and a host of other chronic diseases [10]. However, the role of PUFA in periodontitis is currently inconclusive. Yu Ozaki and other scholars who studied the effects of omega-3 fatty acids intake on osteoclast differentiation and maturation in a mouse model of periodontitis reported that omega-3 fatty acids intake inhibited osteoclast differentiation as well as bone resorption and tissue destruction induced by periodontitis [11]. Hyojin Heo’s meta-analysis also revealed that omega-3 fatty acids intake considerably decreased the depth of the periodontal pocket, improved clinical attachment loss (AL), and reduced the probing bleeding index [12]. However, a study in Finland revealed no statistically significant association between periodontal characteristics and the intake of omega-3 or omega-6 fatty acids or the omega-3/omega-6 fatty acids ratio using data from the Health Survey 2000 in Finland [13]. Considering that most of the early studies were retrospective studies on diet or had small sample sizes, bias might be present. Mendelian randomisation (MR) stands out as a widely used analytical approach employing genetic variation as an instrumental variable. By utilising genetic predictors of risk factors, this method benefits from the natural zrandomisation of alleles during meiosis, ensuring their independent distribution across the population. As a result, MR is generally less susceptible to reverse causality bias and confounding. Moreover, MR is a widely utilised analytical approach that employs genetic variation as an instrumental variable. By utilising genetic predictors of risk factors, this method leverages the natural randomisation of alleles during meiosis, ensuring their independent distribution across the population. Consequently, MR is generally less prone to reverse causality bias and confounding factors [14, 15]. To date, there are no relevant reports on the causal relationship between PUFA concentrations and the risk of developing periodontitis.

Therefore, we aimed to research the association between plasma unsaturated fatty acids concentrations and the risk of developing periodontitis based on the National Health and Nutrition Examination Survey (NHANES) dataset and to assess the causality of that association using large-scale genome-wide association study (GWAS) data under the framework of MR.

## Materials and methods

### Observational research design and data sources

**Fig. 1:**
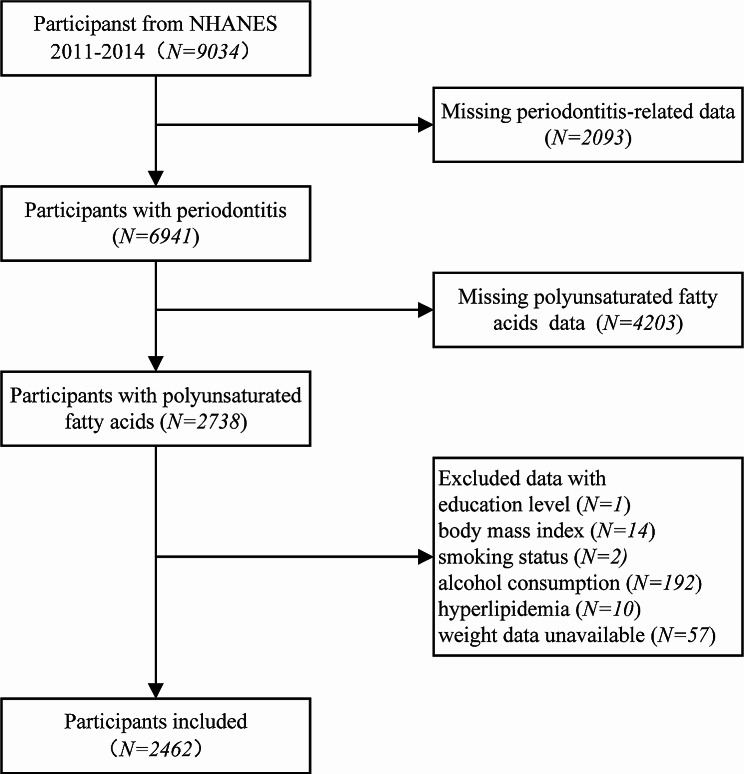
The flowchart of NHANES database study

The NHANES is a nationally representative cross-sectional study conducted under the direction of the National Center for Health Statistics (NCHS), and it was designed to assess the health and nutritional status of the U.S. noninstitutionalized population using a complex, multistage probability sampling design. All data collected from NHANES participants were approved for use in research by the NCHS Ethical Review Board (available on the website https://www.cdc.gov/nchs/nhanes/). The data were acquired from two NHANES cycles (2011–2012, 2013–2014) based on the availability of periodontitis-related data and plasma unsaturated fatty acids concentration data. A total of 9034 participants aged 30 to 80 years were enrolled. A comprehensive periodontal examination was performed on 6941 of these individuals, providing enough information to classify their periodontitis status. Those without information on plasma PUFA concentrations (*n* = 4203), education level (*n* = 1), body mass index (BMI) (*n* = 14), smoking status (*n* = 2), alcohol consumption (*n* = 192), hyperlipidaemia (*n* = 10), or weight data (*n* = 57) were excluded. Ultimately, 2462 participants were included in subsequent analyses. The details of the inclusion and exclusion process are displayed in Fig. 1.

### Periodontal classification

According to the classification of periodontitis by the Centers for Disease Control and Prevention and the American Academy of Periodontology (CDC-AAP), its severity can be categorized into three levels: mild, moderate, and severe, based on probing depth (PD) and clinical AL. Mild periodontitis was defined as having ≥ 2 interproximal sites with ≥ 3 mm clinical AL and ≥ 2 interproximal sites with ≥ 4 mm PD (not on the same tooth) or one site with ≥ 5 mm PD. Moderate periodontitis was defined as having ≥ 2 interproximal sites with PD ≥ 5 mm (not on the same tooth), or ≥ 2 interproximal sites with AL ≥ 4 mm (not on the same tooth). Severe periodontitis was defined as having ≥ 2 interproximal sites with AL ≥ 6 mm (not on the same tooth) and ≥ 1 interproximal site with PD ≥ 5 mm [16]. Participants were classified as not having periodontitis if they did not fall into any of the above categories. In our study, participants were divided into two groups: non-periodontitis and periodontitis group, and those who were diagnosed with mild, moderate, or severe periodontitis were classified into the periodontitis group.

### PUFA subcategories

The detailed procedures for measuring fatty acids in blood are available in the NHANES. Omega-3 and omega-6 PUFA are the two main categories of PUFA for which data are provided in the NHANES database. Among these fatty acids, omega-3 includes alpha-linolenic acid (ALA;18:3n-3), eicosapentaenoic acid (EPA; 20:5n-3), docosapentaenoic acid (n-3 DPA; 22:5n-3), and docosahexaenoic acid (DHA; 22:6n-3); omega-6 includes linoleic acid (LNA; 18:2n-6), gamma-linolenic acid (GLA; 18:3n-6), eicosadienoic acid (EDA; 20:2n-6), homo-gamma-linolenic acid (HGL; 20:3n-6), arachidonic acid (AA; 20:4n-6), docosatetraenoic acid (DTA; 22:4n-6), and docosapentaenoic acid (n-6 DPA; 22:5n-6).

### Definition of covariables

Based on prior literature, we included several potential confounding factors associated with the risk of periodontitis [17]. The demographic characteristics included age, gender, race (non-Hispanic white, non-Hispanic black, Hispanic and other races), and education level. BMI was calculated as weight in kilograms divided by height in metres squared. Participants were categorized as underweight or normal weight (< 25 kg/m^2^), overweight (25–30 kg/m^2^), and obese (> 30.0 kg/m^2^) based on BMI [17]. Education was also divided into three categories: less than high school, high school, and more than high school. Smoking status was classified as never, former, and now. All three levels were classified using 100 cigarettes as nodes: smoked less than 100 cigarettes in a lifetime was classified as “never”, smoking more than 100 cigarettes in a lifetime but currently do not smoke was defined “former”, smoked more than 100 cigarettes in a lifetime and smoking on certain days or every day was defined as “now” [18]. Alcohol consumption status was divided into never (less than 12 drinks in a lifetime), former (≥ 12 drinks in 1 year and no drinks in the past year, or no drinks in the past year but ≥ 12 drinks in a lifetime), mild (defined as an average of ≤ 1 drink per day for women and ≤ 2 drinks per day for men over the past 12 months), moderate (1–3 drinks per day for women and 2–4 drinks per day for men), and heavy (on average, women drank ≥ 4 drinks per day and men drank ≥ 5 drinks per day in the past 12 months) [19]. Hypertension was defined by a self-reported history of hypertension, the use of antihypertensive medications, a mean systolic blood pressure ≥ 140 mmHg, and/or a mean diastolic blood pressure ≥ 90 mmHg. Diabetes was defined by any of the following: HbA1c ≥ 6.5%, 2-hour plasma glucose ≥ 200 mg/dL after a 75-g glucose load (OGTT), fasting plasma glucose ≥ 126 mg/dL, and self-reported diagnosis of diabetes, or any self-reported insulin or use of other diabetes medications [20]. A total cholesterol concentration ≥ 200 mg/dL, triglyceride concentration ≥ 150 mg/dL, male HDL concentration < 40 mg/dL, female HDL concentration < 50 mg/dL, and low-density lipoprotein cholesterol concentration ≥ 130 mg/dL were the criteria for hyperlipidaemia. Additionally, the use of cholesterol-lowering drugs was also defined as hyperlipidaemia [21].

### Statistical analysis

The SDMVPSU and SDMVSTRA programs were used for the complex survey design of the NHANES, and WTFAS2YR was used to weight all analyses for nationally representative estimates. Categorical variables and medians (the 25% and 75% quartiles) were used to display the characteristics of the participants. The Rao–Scott modified chi-square test and weighted Mann‒Whitney U test were used to compare the differences between the two groups. Then, weighted logistic regression models were performed to evaluate the association between PUFA concentrations and the risk of periodontitis. Model 1 did not include any covariable; Model 2 was adjusted for age, gender, and race; and Model 3 was based on Model 2 and adjusted for smoking status, alcohol consumption, education, BMI, diabetes, hypertension, and hyperlipidaemia. The R package “rms” was applied to perform restricted cubic splines (RCS) to analyse the nonlinear relationship between PUFA concentrations and the risk of periodontitis. E values were utilized to evaluate the robustness of potential unmeasured factors. Additionally, differences in confounders between the two groups were balanced through 1:1 propensity score matching (PSM). Subsequently, the post-PSM data were reanalysed to further validate the accuracy of the findings. All data analyses were performed using R software (version 4.3.1). *P* value < 0.05 was considered to indicate statistical significance.

### MR analysis

#### Sources of plasma fatty acids and chronic periodontitis phenotypes

The GWAS datasets for omega-3 fatty acids, omega-6 fatty acids, and the omega-6/omega-3 fatty acids ratio were obtained from the IEU database (https://gwas.mrcieu.ac.uk/). These datasets, created in 2020, involved an analysis of 114,999 individuals of European descent, resulting in 12,321,875 SNPs undergoing GWAS analysis. For chronic periodontitis, the GWAS dataset was acquired from the FinnGen database (https://www.finngen.fi/en/access_results), with diagnoses rigorously classified according to the International Classification of Diseases, Tenth Revision (ICD-10 codes K05.30, K05.31). This dataset included 4,784 patients with periodontitis and 272,252 control participants.

#### Two-sample bidirectional MR

In our initial approach to forwards MR, we selected single-nucleotide polymorphisms (SNPs) that showed a strong association with the exposure variable (*P* < 5e-8). To address linkage disequilibrium, SNPs within close physical proximity (< 10,000 kb) or those with an R^2^ < 0.001 were excluded [22]. Furthermore, a Steiger filtering test was conducted to eliminate SNPs from the instrumental variables that exhibited a causal direction opposite to the one under investigation, thereby minimizing the influence of reverse causality between the exposure and outcome [23]. Additionally, palindromic SNPs with unclear directional impact were removed. To ascertain the robustness of our instrumental variables, we calculated the F-statistic for all SNPs, discarding those with F < 10 [24]. The criteria for selecting instrumental variables in the reverse MR analysis were consistent with those in the forwards MR analysis. However, due to the limited number of SNPs meeting the *P* < 5e-8 threshold for chronic periodontitis, we adjusted the significance level to *P* < 1e-5 while still computing the F value to confirm the reliability of the instrumental variables. To assess MR causality, we utilized three distinct methodologies: the inverse-variance weighting (IVW), MR-Egger, and weighted median (WM) methods. The IVW method was implemented with a random-effects model when heterogeneity was present in the results, providing a more nuanced analysis, whereas a fixed-effects model was employed when no heterogeneity was detected.

#### Sensitivity analysis

To ensure the stability of our findings, comprehensive sensitivity analyses were performed. Cochran’s Q test was applied to identify heterogeneity among the instrumental variable SNPs, which guided the selection of an appropriate IVW method contingent on detected heterogeneity levels [25]. The MR-Egger regression model served to detect potential horizontal pleiotropy within the MR framework. The identification of horizontal pleiotropy suggests possible confounding in the causal effect estimates, which potentially compromised the reliability of the study results [26]. Furthermore, the leave-one-out analysis, presented visually herein, was instrumental for the identification of any significant outlier SNPs within the instrumental variables that could markedly influence the causal inference [27].

#### Statistical analysis

Bidirectional two-sample MR analysis was conducted using the package “TwoSampleMR” (version 0.5.6) in R software (version 4.3.1).

## Result

### Baseline characteristics

**Fig. 2:**
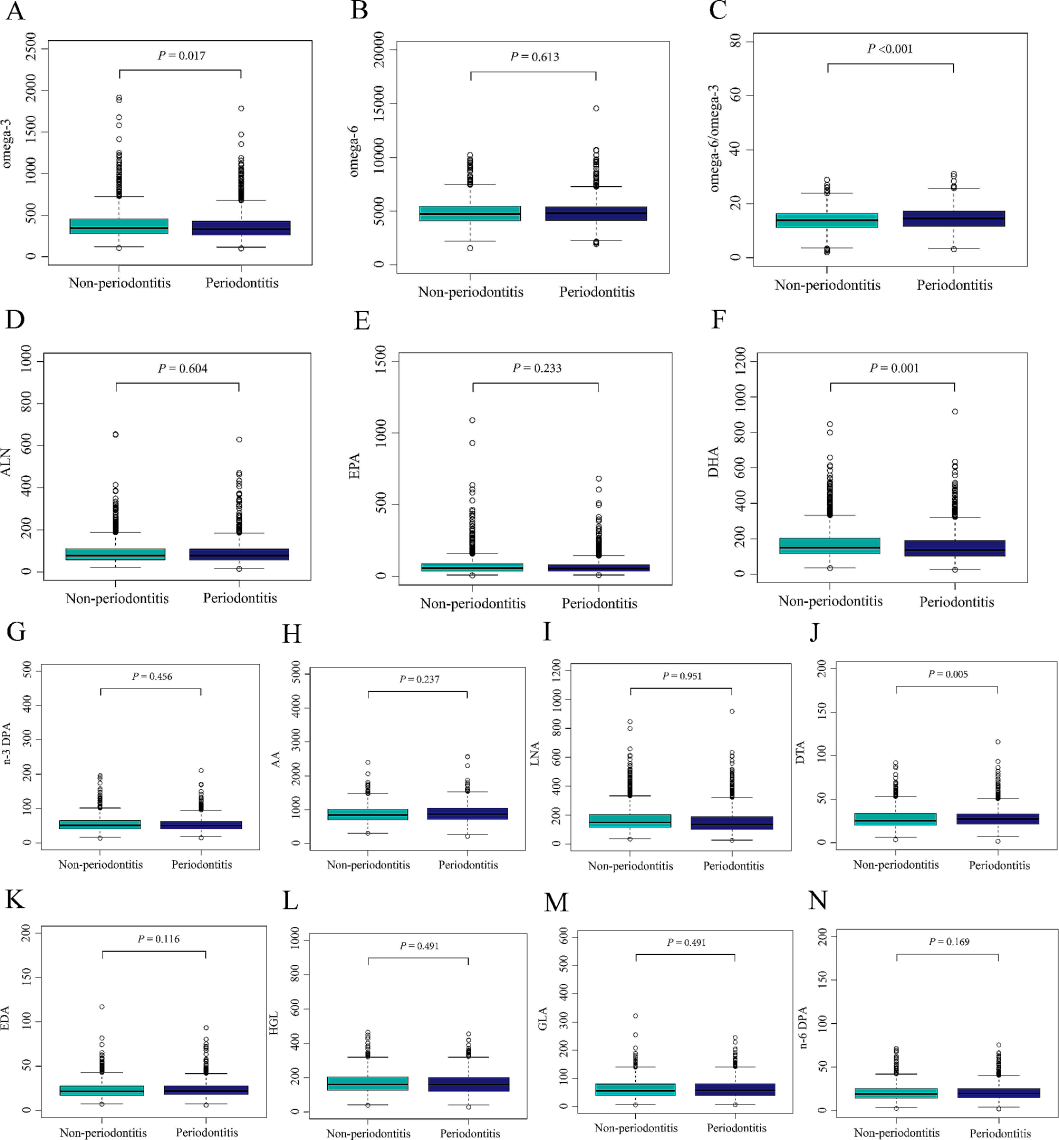
The difference in PUFA between the periodontitis and non-periodontitis group

Table 1 displays that our study included 2462 individuals from the NHANES, comprising 1139 periodontitis and 1323 non-periodontitis participants. There were significant differences in age, gender, race, education level, smoking status, alcohol consumption, BMI, diabetes, and hypertension between the two groups (*P* < 0.05). In addition, the difference in PUFA between the two groups is shown in Fig. 2. Compared with those in the non-periodontitis group, participants with periodontitis had higher concentrations of DTA, while the concentrations of DHA and omega-3 fatty acids and the omega-6/omega-3 fatty acids ratio were lower (*P* < 0.05). The weighted medians and percentages (25% and 75%, respectively) of PUFA and their subcategories among all participants are listed in Table S1: omega-3: 337 (271, 439); LNA: 77 (58, 109); EPA: 56 (38, 84); n-3 DPA: 51 (41, 64); DHA: 145 (111, 197); omega-6: 4,762 (4,128, 5,437); LNA: 3,600 (3,050, 4,138); GLA: 57 (40, 80); EDA: 22 (18, 28); HGL: 161 (126, 203); AA: 856 (702, 1,030); DTA: 26 (20, 33); n-6 DPA: 20 (15, 26); and omega-6/omega-3: 14.2 (11.5, 16.7).

**Table 1:** Characteristics of participants in NHANES 2011–2014 according to periodontitis status

Characteristic	Overall (*N* = 2462)	Non-periodontitis group (*N* = 1323)	Periodontitis group (*N* = 1139)	*P*
**Age (years)**	50 (40, 61)	46 (38, 59)	55 (44, 64)	< 0.001
**Gender**				< 0.001
Female	1,228 (49.50)	752 (54.49)	476 (41.24)	
Male	1,234 (50.50)	571 (45.51)	663 (58.76)	
**Race**				< 0.001
Non-Hispanic White	1,066 (69.56)	671 (75.60)	395 (59.59)	
Other races	649 (14.38)	328 (12.11)	321 (15.77)	
Non-Hispanic Black	498 (10.64)	198 (7.53)	300 (18.15)	
Other Hispanic	249 (5.42)	126 (4.77)	123 (6.49)	
**Education**				< 0.001
Less than high school	504 (14.40)	166 (8.01)	338 (24.97)	
High school	499 (19.35)	219 (16.09)	280 (24.74)	
More than high school	1,459 (66.25)	938 (75.89)	521 (50.29)	
**BMI**				0.043
Underweight/normal	661 (24.75)	360 (24.76)	301 (24.75)	
Overweight	852 (36.09)	477 (38.45)	375 (32.17)	
Obese	949 (39.15)	486 (36.79)	463 (43.06)	
**Smoking status**				< 0.001
Never	1,400 (56.78)	857 (64.11)	543 (44.66)	
Former	624 (26.09)	313 (24.58)	311 (28.57)	
Now	438 (17.13)	153 (11.30)	285 (26.77)	
**Alcohol consumption**				0.002
Never	341 (10.02)	164 (9.16)	177 (11.42)	
Former	544 (18.92)	255 (16.38)	289 (23.12)	
Mild	784 (35.38)	460 (38.71)	324 (29.88)	
Moderate	356 (17.03)	229 (19.07)	127 (13.66)	
Heavy	437 (18.65)	215 (16.68)	222 (21.92)	
**Diabetes**				< 0.001
No	1,945 (83.65)	1,124 (88.63)	821 (75.42)	
Yes	517 (16.35)	199 (11.37)	318 (24.58)	
**Hypertension**				0.002
No	1,365 (58.56)	817 (62.60)	548 (51.89)	
Yes	1,097 (41.44)	506 (37.40)	591 (48.11)	
**Hyperlipidaemia**				0.245
No	640 (25.41)	363 (26.43)	277 (23.72)	
Yes	1,822 (75.49)	960 (73.57)	862 (76.28)	
**Mean AL**	1.43 (1.09, 1.93)	1.18 (0.97, 1.44)	2.08 (1.69, 2.79)	< 0.001
**Mean PD**	1.28 (1.01, 1.60)	1.10 (0.89, 1.32)	1.65 (1.36, 2.06)	< 0.001

### The association between PUFA concentrations and the risk of periodontitis

As illustrated in Table 2, we analysed the association between PUFA concentrations and the risk of periodontitis through weighted logistic regression analysis. There was a significant negative relationship between the risk of periodontitis and omega-3 fatty acids intake in all the models (*P* < 0.05). The risk of periodontitis decreased with increasing omega-3 content. However, the omega-6/omega-3 fatty acids ratio was positively associated with periodontitis risk (*P* < 0.05). There was no clear relationship between the risk of periodontitis and omega-6 fatty acids concentrations. Furthermore, we performed weighted regression on all subcategories to explore the association between the risk of periodontitis and the concentrations of fatty acids in the omega-3 and omega-6 subcategories (Table S2, Table S3). The results demonstrated a substantial negative association between DHA concentrations and the risk of periodontitis (*P* < 0.05). Furthermore, except for DTA concentration, which was a risk factor for periodontitis in Models 1 and 2, no other subcategories were found to be significantly associated with periodontitis.

**Table 2:** Weighted logistic regression analysis of the association between PUFA and periodontitis

Characteristic	Model 1		Model 2		Model 3	
	OR (95% CI)	*P*	OR (95% CI)	*P*	OR (95% CI)	*P*
**Omega-3**						
Q1	*Ref*		*Ref*		*Ref*	
Q2	0.75 (0.57, 0.97)	0.030	0.68 (0.48, 0.97)	0.034	0.75 (0.51, 1.10)	0.127
Q3	0.77 (0.56, 1.05)	0.091	0.60 (0.42, 0.86)	0.008	0.67 (0.45, 1.00)	0.052
Q4	0.66 (0.49, 0.88)	0.006	0.48 (0.35, 0.66)	< 0.001	0.59 (0.41, 0.85)	0.008
*P* for trend		0.017		< 0.001		0.008
**Omega-6**						
Q1	*Ref*		*Ref*		*Ref*	
Q2	1.00 (0.76, 1.32)	0.988	1.17 (0.88, 1.57)	0.270	1.16 (0.82, 1.63)	0.363
Q3	1.18 (0.85, 1.63)	0.313	1.38 (0.97, 1.96)	0.074	1.45 (1.02, 2.07)	0.038
Q4	0.97 (0.74, 1.27)	0.819	1.05 (0.79, 1.41)	0.710	1.10 (0.78, 1.55)	0.545
*P* for trend		0.008		0.510		0.326
**Omega-6/omega-3**						
Q1	*Ref*		*Ref*		*Ref*	
Q2	0.98 (0.72, 1.35)	0.910	1.23 (0.87, 1.74)	0.220	1.12 (0.75, 1.67)	0.560
Q3	1.05 (0.80, 1.37)	0.738	1.51 (1.14, 1.99)	0.006	1.26 (0.91, 1.74)	0.143
Q4	1.57 (1.21, 2.04)	0.001	2.59 (1.92, 3.48)	< 0.001	1.88 (1.37, 2.56)	0.001
*P* for trend		0.002		< 0.001		0.001

Note: Model 1: Unadjusted model; Model 2: Adjusted for age, gender, race; Model 3: Adjusted for age, gender, race, smoking status, alcohol consumption, education, BMI, diabetes, hypertension, and hyperlipidaemia. (OR = Odds Ratio, CI = Confidence Interval)

### RCS

**Fig. 3:**
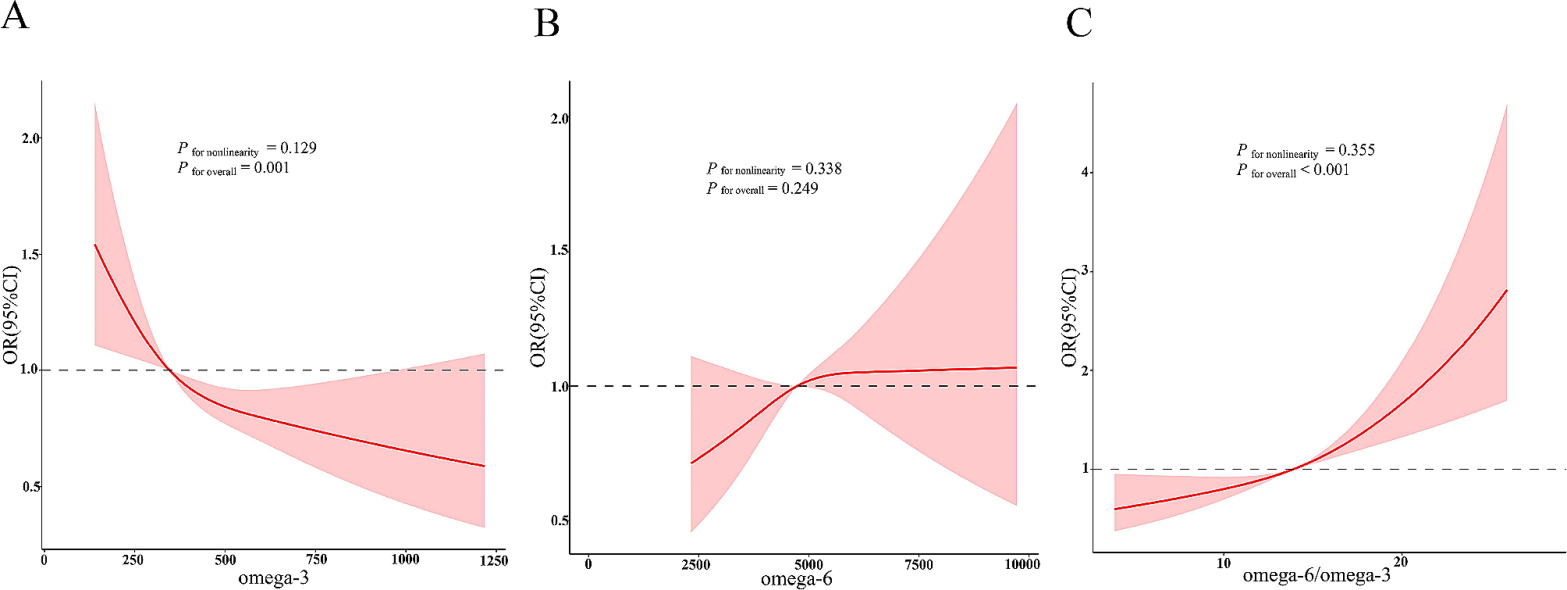
Restricted cubic spline curves of PUFA. Adjusted for age, gender, race, smoking status, alcohol consumption, education level, BMI, diabetes, hypertension, and hyperlipidaemia. **A**: RCS of omega-3; **B**: RCS of omega-6; **C**: RCS of omega-6/omega-3

The RCS results demonstrated that the concentrations of omega-3 and omega-6 fatty acids and the omega-6/omega-3 fatty acids ratio had no nonlinear relationship with the risk of periodontitis (*P* for nonlinearity > 0.05), but there was a dose-dependent relationship of the concentration of omega-3 fatty acids and the omega-6/omega-3 fatty acids ratio with the risk of periodontitis (*P* < 0.05) (Fig. 3).

### Sensitivity analysis

As demonstrated in Table S4, the E values suggested that our results are robust, provided that there are no unmeasured confounders whose relative risks exceed the E values associated with PUFA. Additionally, Table S5 illustrates that the differences in covariates between the two groups were controlled following PSM. Moreover, Figure S1 and Tables S6-S8 demonstrate that the results after PSM were consistent with those prior to matching.

### Bidirectional causal relationships between plasma fatty acids and chronic periodontitis

**Fig. 4:**
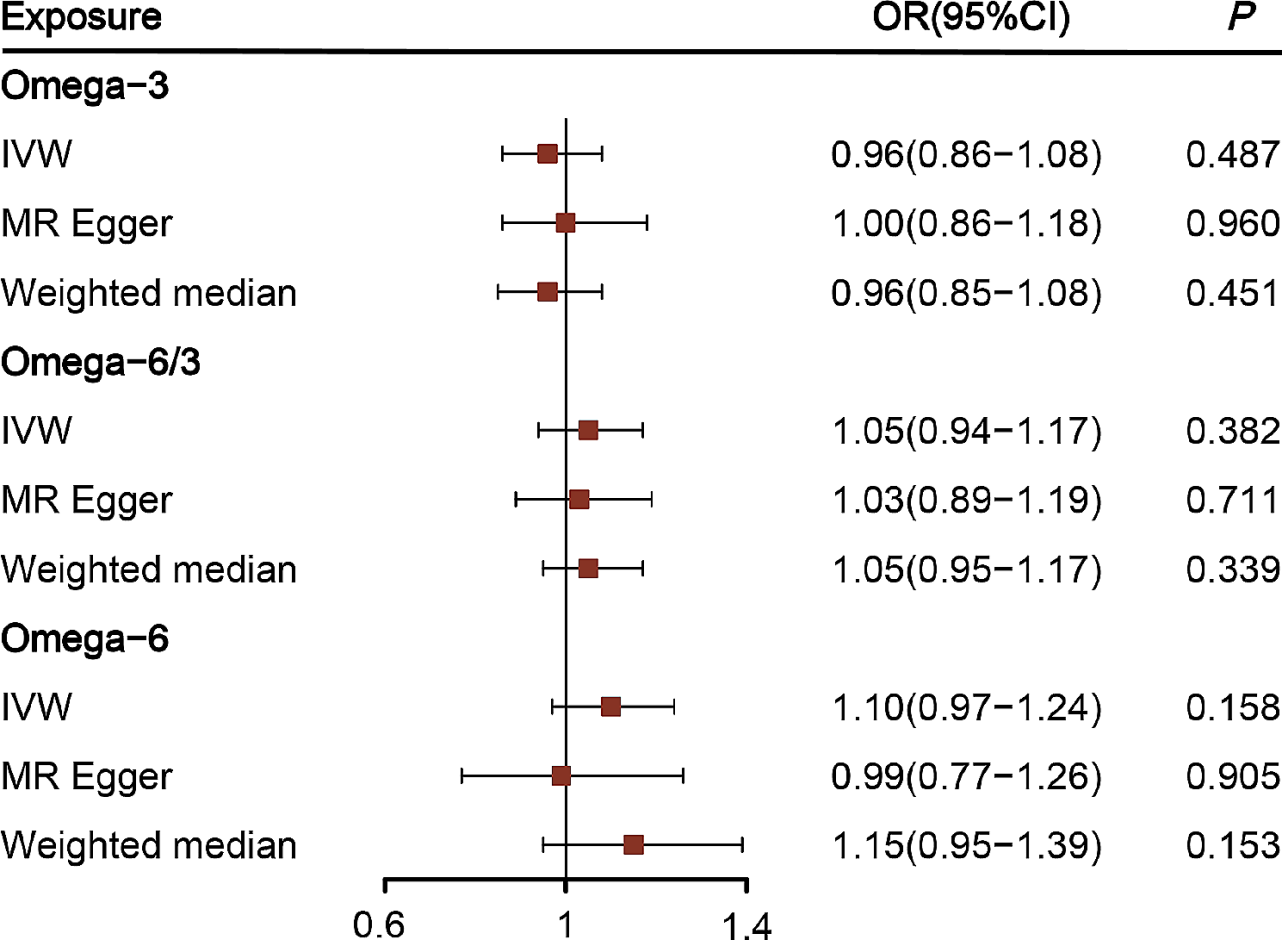
The forward Mendelian randomization causal assessment of the impact of PUFA on periodontitis

In the forwards MR analysis, 48, 34, and 52 SNPs were identified as instrumental variables for omega-3 fatty acids, the ratio of omega-6 to omega-3 fatty acids, and omega-6 fatty acids, respectively. The forwards MR findings revealed no causal relationship between plasma concentrations of these fatty acids and periodontitis risk (Fig. 4). Moreover, in the reverse MR, 24 SNPs were qualified and included as instrumental variables for chronic periodontitis, showing that chronic periodontitis does not causally influence the levels of omega-3 fatty acids, the ratio of omega-6 to omega-3 fatty acids, or omega-6 fatty acids (Figure S2). Sensitivity analyses were performed for both directions of MR analysis (Table S9-S10). Cochran’s Q test indicated heterogeneity among the instrumental variables for omega-3 fatty acids, necessitating the adoption of the IVW random-effects model (Table S9). Moreover, MR-Egger regression suggested that no horizontal pleiotropy affected any of the MR outcomes. Leave-one-out analysis further confirmed the absence of any significant outlier SNPs that could skew the causal estimates (Figure S3).

## Discussion

Many researchers are now shedding light on host-modulated periodontitis treatments, which are thought to be a viable therapeutic strategy. In this context, we comprehensively investigated the relationship between PUFA concentrations and the risk of periodontitis based on a large-scale observational study dataset and used MR to assess the possible causality between the two using a significant amount of genetic data.

The negative associations between omega-3 fatty acids levels and the risk of periodontitis and between omega-3 fatty acids levels and periodontal parameters were consistent with the findings of most studies [28, 29]. Stańdo-Retecka, M. et al. reported that compared to patients in the control group, patients treated with omega-3 PUFA demonstrated significantly lower rates of bleeding on probing (BOP), greater AL gains, and an increased number of closed pockets at the three-month mark [30]. The anti-inflammatory, antibacterial, and inflammatory bone loss-regulating properties of omega-3 might be the primary mechanisms by which it regulates periodontitis [31–33]. Furthermore, EPA and DHA, as the most bioactive fatty acids among omega-3 PUFA, could be oxidized to form a group of oxylipids called specialised pro-resolving mediators (SPM), which have anti-inflammatory effects and are reported to regulate leukocyte infiltration, blocks interleukin (IL)-1-induced activation of nuclear factor kappa-light chain enhancer of activated B cells (NF-κB) and reduces the expression of pro-inflammatory cytokines [34, 35]. To this end, we further explored the associations between the risk of periodontitis and EPA, DHA, and other omega-3 subcategories. However, only DHA was negatively associated with the risk of periodontitis, while EPA and other omega-3 subclasses had no significant association. Studies have reported that the intake of fish oil containing 54% DHA could increase the phagocytic activity of neutrophils and monocytes by 62% and 145%, respectively, while these changes were not observed in fish oil rich in EPA [36, 37]. The differences in phagocytic activity might be the reason for their significantly different risks of periodontal disease.

The proinflammatory effect of omega-6 PUFA is well known [38–40]. In particular, AA is metabolized through the cyclooxygenase and lipoxygenase pathways, producing eicosanoids such as prostaglandins, thromboxanes, and leukotrienes, which play a significant role in promoting inflammation [41]. Additionally, the research by Sztolsztener K and colleagues suggested that changes in AA levels could serve as early indicators of irreversible progression of inflammation and nonalcoholic fatty liver disease [42]. However, our results showed that except for DTA, which is associated with an increased risk of developing periodontal disease, omega-6 and other subclasses were not significantly associated with the risk of periodontitis. Notably, we found that the omega-6/omega-3 fatty acids ratio was positively associated with the risk of periodontitis, which is consistent with multiple studies reporting that a lower ratio of omega-6 to omega-3 fatty acids intake can improve health and reduce the risk of developing many chronic diseases [43, 44]. This may be related to DHA, EPA, or DPA competing with arachidonic acids as substrates for COX and LOX, thereby reducing the production of inflammatory eicosanoids. However, unlike the recommended ratio of omega-6 to omega-3 fatty acids in the diet, which is 4:1, we found that the risk of periodontitis only occurs when the ratio is greater than 10. This might be because the participants in our sample were American and the Western diet is known for having a high consumption of omega-6 fatty acids and a low intake of omega-3 fatty acids. Furthermore, there might be more than one optimal pattern for the intake of n-6 and n-3 fatty acids, necessitating consideration of genetics, developmental stages, and social environments to tailor the best recommendations for specific individuals [45]. At the same time, the potential causal association between the two was analysed through MR of large-scale genetic data. The results of our MR analysis did not support a causal association between unsaturated fatty acids and periodontitis risk. More specifically, our results indicated that increased plasma concentrations of PUFA were not associated with the risk of periodontitis.

This study has several advantages. First, plasma PUFA concentrations can serve as objective biomarkers of PUFA intake, avoiding the well-known bias in self-reported dietary intake assessments [46]. Second, compared with observational studies, MR analysis significantly reduces biases such as reverse causality and confounding factors. Importantly, MR analysis enabled the assessment of causal associations between plasma PUFA concentrations and the risk of periodontitis. However, some limitations need to be considered when interpreting our findings. First, the study sample consisted of individuals of American and European ancestry, which may limit the generalizability of the results to other ethnic groups [47, 48]. Second, periodontitis samples may exhibit heterogeneity due to variations in the definitions of periodontal disease cases, potentially influencing the reliability and generalizability of our conclusions. Additionally, our study did not include key metabolites of PUFA, such as oxylipins and prostaglandins, which may impact the interpretability and applicability of our findings. Therefore, large-scale, multisample prospective studies may be required to further investigate the association between PUFA concentrations and the risk of periodontitis in the future.

## Conclusion

Our study suggests that there is no evidence indicating that elevated PUFA levels might be beneficial for preventing periodontitis. However, these findings need to be confirmed by large-scale, multisample prospective studies in the future.

### Electronic supplementary material

Below is the link to the electronic supplementary material.

Supplementary Material 1

## Data Availability

The dataset(s) supporting the conclusions of this article are available in the NHANES database: https://www.cdc.gov/nchs/nhanes/; IEU: https://gwas.mrcieu.ac.uk/; FinnGen: https://www.finngen.fi/en/access_results.
