# Supplementary material for: The association between polyunsaturated fatty acids and periodontitis: NHANES 2011–2014 and Mendelian randomisation analysis
Source: Lipids Health Dis. 2024 Jun 4;23:168. doi: 10.1186/s12944-024-02159-0 (PMC11149223; doi:10.1186/s12944-024-02159-0)
Supplement: Supplementary file 1 — Supplementary Material 1 [file 12944_2024_2159_MOESM1_ESM.docx]

Supplementary materials


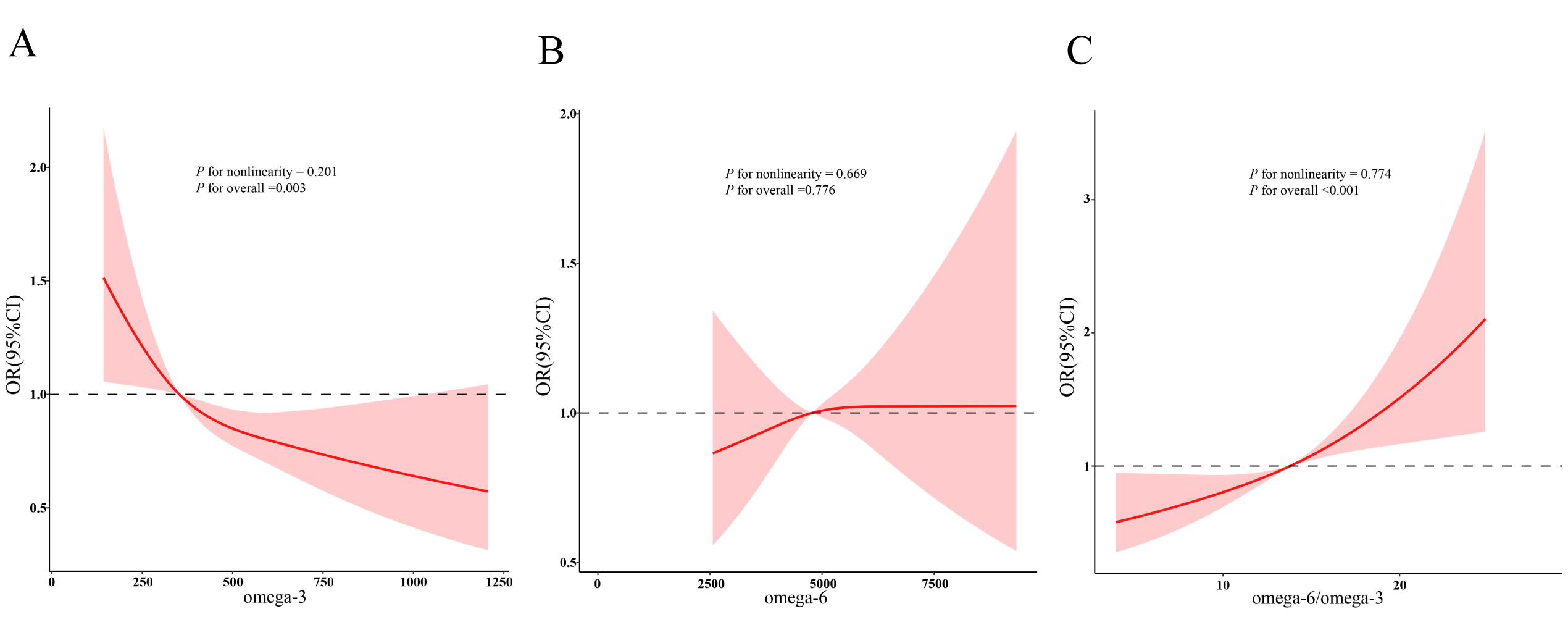


**Figure S1:** Restricted cubic spline curves of PUFA after PSM. Adjusted for age, gender, race, smoking status, alcohol consumption, education, BMI, diabetes, hypertension, and hyperlipidaemia. A: RCS of omega-3; B: RCS of omega-6; C: RCS of omega-6/omega-3.


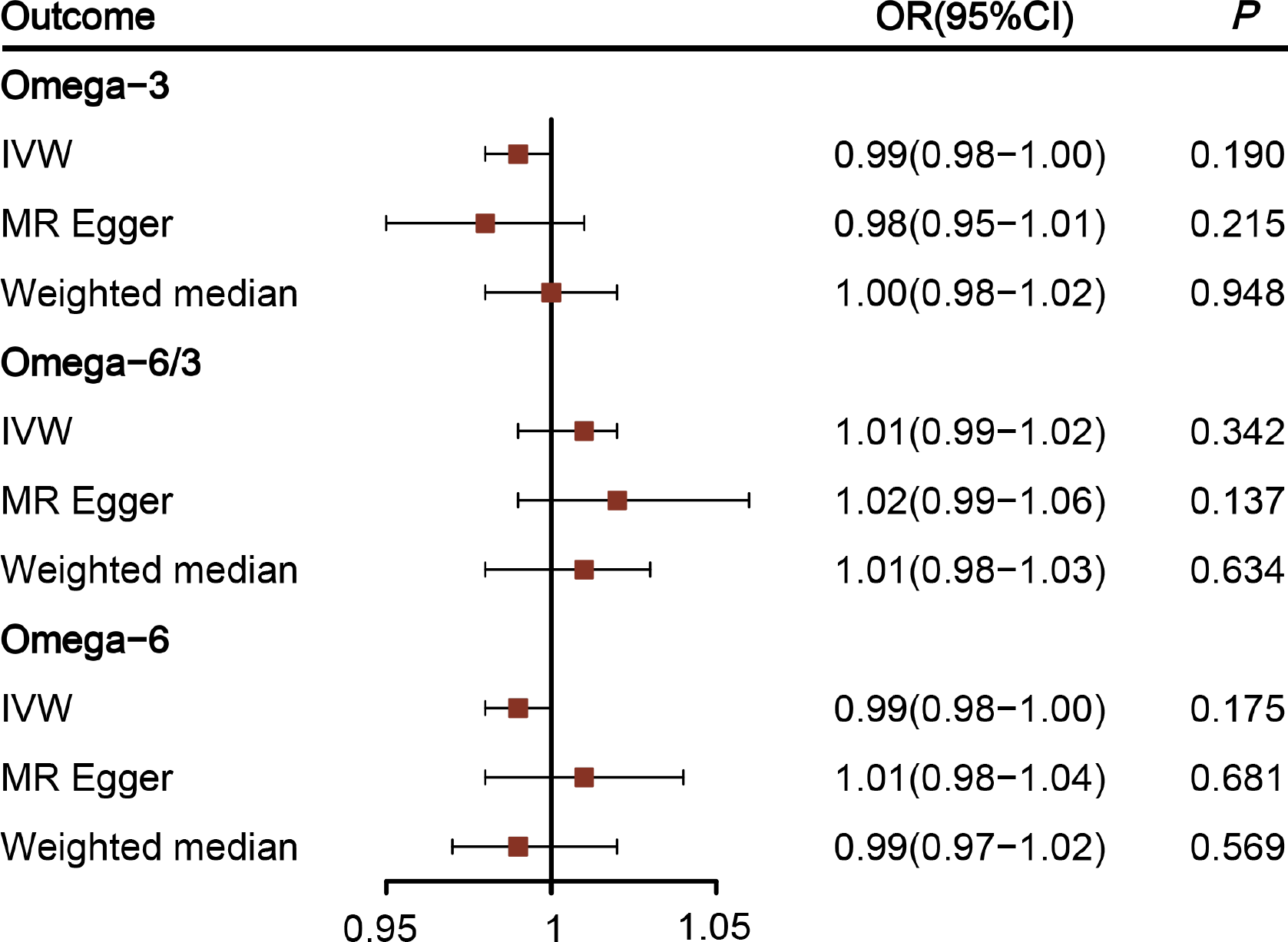


**Figure S2:** The reverse Mendelian randomisation causal assessment of the impact of periodontitis on PUFA.

s


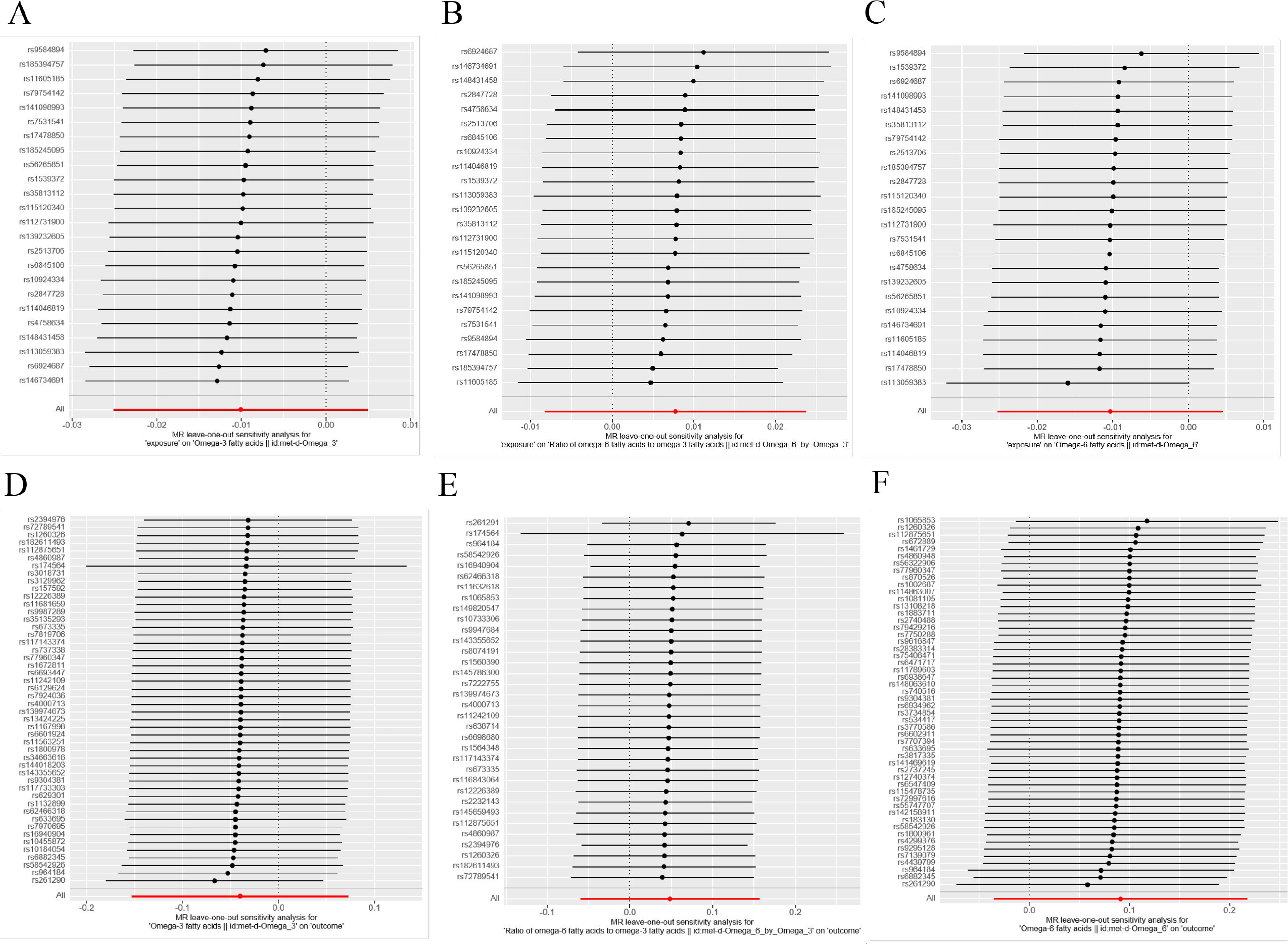


**Figure S3:** Leave-one-out sensitivity analysis. A: Periodontitis on omega-3; B: Periodontitis on the ratio of omega-6/omega-3; C: Periodontitis on omega-6; D: Omega-3 on periodontitis; E: The ratio of omega-6/omega-3 on periodontitis; F: Omega-6 on periodontitis.

**Table S1:** The difference in PUFA between the periodontitis and non-periodontitis group.

| **Characteristic** | **Overall (N = 2462)** | **Non-periodontitis group  (N = 1323)** | **Periodontitis group  (N = 1139)** | ***P*** |
| --- | --- | --- | --- | --- |
| **Omega-3** | 337 (271, 439) | 345 (276, 455) | 331 (262, 427) | 0.017 |
| **Omega-6** | 4,762 (4,128, 5,437) | 4,753 (4,113, 5,454) | 4,810 (4,137, 5,400) | 0.613 |
| **Omega-6/omega-3** | 14.2 (11.5, 16.7) | 14.0 (11.3, 16.4) | 14.6 (11.7, 17.3) | <0.001 |
| **ALN** | 77 (58, 109) | 77 (58, 110) | 77 (57, 108) | 0.604 |
| **EPA** | 56 (38, 84) | 56 (38, 86) | 55 (38, 81) | 0.233 |
| **n-3 DPA** | 51 (41, 64) | 51 (41, 65) | 50 (41, 63) | 0.456 |
| **DHA** | 145 (111, 197) | 149 (116, 203) | 136 (103, 190) | 0.001 |
| **LNA** | 3,600 (3,050, 4,138) | 3,600 (3,050, 4,150) | 3,620 (3,070, 4,110) | 0.951 |
| **GLA** | 57 (40, 80) | 56 (40, 80) | 58 (41, 81) | 0.182 |
| **EDA** | 22 (18, 28) | 22 (17, 28) | 22 (18, 28) | 0.116 |
| **HGL** | 161 (126, 203) | 161 (127, 204) | 161 (122, 201) | 0.491 |
| **AA** | 856 (702, 1,030) | 848 (698, 1,010) | 873 (713, 1,040) | 0.237 |
| **DTA** | 26 (20, 33) | 25 (20, 33) | 27 (21, 33) | 0.005 |
| **n-6 DPA** | 20 (15, 26) | 19 (15, 26) | 20 (15, 25) | 0.169 |

**Table S2**: Weighted logistic regression analysis of the associations between omega-3 subclass fatty acids and periodontitis.

| **Characteristic** | **Model 1** | |  | **Model 2** | |  | **Model 3** | |
| --- | --- | --- | --- | --- | --- | --- | --- | --- |
|  | **OR (95% CI)** | ***P*** |  | **OR (95% CI)** | ***P*** |  | **OR (95% CI)** | ***P*** |
| **EPA** |  |  |  |  |  |  |  |  |
| Q1 | *Ref* |  |  | *Ref* |  |  | *Ref* |  |
| Q2 | 1.03 (0.80, 1.34) | 0.810 |  | 1.00 (0.78, 1.28) | 0.992 |  | 1.11 (0.83, 1.50) | 0.447 |
| Q3 | 1.06 (0.84, 1.35) | 0.591 |  | 0.98 (0.79, 1.22) | 0.844 |  | 1.10 (0.82, 1.49) | 0.489 |
| Q4 | 0.85 (0.62, 1.17) | 0.309 |  | 0.72 (0.51, 1.02) | 0.064 |  | 0.93 (0.63, 1.38) | 0.694 |
| *P* for trend |  | 0.329 |  |  | 0.052 |  |  | 0.689 |
| **ALN** |  |  |  |  |  |  |  |  |
| Q1 | *Ref* |  |  | *Ref* |  |  | *Ref* |  |
| Q2 | 0.85 (0.60, 1.19) | 0.330 |  | 0.91 (0.64, 1.29) | 0.572 |  | 0.98 (0.64, 1.49) | 0.914 |
| Q3 | 0.98 (0.72, 1.34) | 0.905 |  | 0.94 (0.66, 1.33) | 0.708 |  | 0.98 (0.66, 1.47) | 0.932 |
| Q4 | 0.90 (0.64, 1.26) | 0.518 |  | 0.88 (0.62, 1.27) | 0.491 |  | 0.92 (0.59, 1.44) | 0.682 |
| Q1 |  | 0.729 |  |  | 0.544 |  |  | 0.691 |
| **n-3 DPA** |  |  |  |  |  |  |  |  |
| Q1 | *Ref* |  |  | *Ref* |  |  | *Ref* |  |
| Q2 | 1.21 (0.87, 1.68) | 0.248 |  | 1.15 (0.79, 1.67) | 0.445 |  | 1.07 (0.75, 1.52) | 0.685 |
| Q3 | 1.09 (0.80, 1.47) | 0.581 |  | 0.99 (0.75, 1.30) | 0.936 |  | 0.91 (0.65, 1.27) | 0.548 |
| Q4 | 0.89 (0.68,1.16) | 0.370 |  | 0.78 (0.57, 1.06) | 0.103 |  | 0.76 (0.53, 1.07) | 0.105 |
| *P* for trend |  | 0.276 |  |  | 0.053 |  |  | 0.071 |
| **DHA** |  |  |  |  |  |  |  |  |
| Q1 | *Ref* |  |  | *Ref* |  |  | *Ref* |  |
| Q2 | 0.59 (0.44, 0.79) | <0.001 |  | 0.53 (0.36, 0.78) | 0.002 |  | 0.60 (0.37, 0.95) | 0.034 |
| Q3 | 0.60 (0.42, 0.85) | 0.006 |  | 0.44 (0.29, 0.67) | <0.001 |  | 0.55 (0.34, 0.88) | 0.018 |
| Q4 | 0.55 (0.39, 0.78) | 0.001 |  | 0.34 (0.23, 0.50) | <0.001 |  | 0.47 (0.32, 0.70) | 0.001 |
| *P* for trend |  | 0.002 |  |  | <0.001 |  |  | 0.002 |

**Note**: Model 1: Unadjusted model; Model 2: Adjusted for age, gender, race; Model 3: Adjusted for age, gender, race, smoking status, alcohol consumption, education BMI, diabetes, hypertension, and hyperlipidaemia. OR = Odds Ratio, CI = Confidence Interval.

**Table S3**: Weighted logistic regression analysis of the associations between omega-6 subclass fatty acids and periodontitis.

| **Characteristic** | **Model 1** | |  | **Model 2** | |  | **Model 3** | |
| --- | --- | --- | --- | --- | --- | --- | --- | --- |
|  | **OR (95% CI)** | ***P*** |  | **OR (95% CI)** | ***P*** |  | **OR (95% CI)** | ***P*** |
| **LNA** |  |  |  |  |  |  |  |  |
| Q1 | *Ref* |  |  | *Ref* |  |  | *Ref* |  |
| Q2 | 1.00 (0.70, 1.42) | 0.997 |  | 1.15 (0.79, 1.67) | 0.454 |  | 1.18 (0.79, 1.76) | 0.377 |
| Q3 | 1.15 (0.85, 1.57) | 0.353 |  | 1.33 (0.91, 1.94) | 0.139 |  | 1.40 (0.94, 2.08) | 0.087 |
| Q4 | 0.97 (0.71, 1.33) | 0.848 |  | 1.09 (0.77, 1.55) | 0.620 |  | 1.20 (0.81, 1.80) | 0.332 |
| *P* for trend |  | 0.908 |  |  | 0.446 |  |  | 0.212 |
| **GLA** |  |  |  |  |  |  |  |  |
| Q1 | *Ref* |  |  | *Ref* |  |  | *Ref* |  |
| Q2 | 0.98 (0.75, 1.28) | 0.904 |  | 0.98 (0.74, 1.31) | 0.912 |  | 0.90 (0.65, 1.24) | 0.474 |
| Q3 | 1.23 (0.93, 1.63) | 0.141 |  | 1.26 (0.95, 1.68) | 0.107 |  | 1.12 (0.80, 1.57) | 0.482 |
| Q4 | 1.09 (0.77, 1.55) | 0.621 |  | 1.22 (0.84, 1.78) | 0.275 |  | 1.05 (0.65, 1.71) | 0.826 |
| *P* for trend |  | 0.356 |  |  | 0.912 |  |  | 0.596 |
| **EDA** |  |  |  |  |  |  |  |  |
| Q1 | *Ref* |  |  | *Ref* |  |  | *Ref* |  |
| Q2 | 1.57 (1.13, 2.16) | 0.008 |  | 1.58 (1.12, 2.23) | 0.011 |  | 1.56 (1.05, 2.31) | 0.030 |
| Q3 | 1.45 (1.11, 1.89) | 0.008 |  | 1.33 (0.98, 1.79) | 0.062 |  | 1.29 (0.90, 1.84) | 0.148 |
| Q4 | 1.32 (0.95, 1.82) | 0.094 |  | 1.28 (0.90, 1.81) | 0.160 |  | 1.14 (0.76, 1.71) | 0.505 |
| *P* for trend |  | 0.115 |  |  | 0.278 |  |  | 0.749 |
| **HGL** |  |  |  |  |  |  |  |  |
| Q1 | *Ref* |  |  | *Ref* |  |  | *Ref* |  |
| Q2 | 0.84 (0.63, 1.12) | 0.232 |  | 0.92 (0.68, 1.22) | 0.538 |  | 0.94 (0.70, 1.26) | 0.637 |
| Q3 | 0.96 (0.70, 1.33) | 0.801 |  | 1.17 (0.83, 1.65) | 0.366 |  | 1.13 (0.78, 1.65) | 0.485 |
| Q4 | 0.89 (0.64, 1.22) | 0.442 |  | 1.19 (0.88, 1.61) | 0.240 |  | 1.03 (0.72, 1.45) | 0.878 |
| *P* for trend |  | 0.669 |  |  | 0.164 |  |  | 0.656 |
| **AA** |  |  |  |  |  |  |  |  |
| Q1 | *Ref* |  |  | *Ref* |  |  | *Ref* |  |
| Q2 | 1.01 (0.77, 1.33) | 0.915 |  | 0.95 (0.68, 1.30) | 0.721 |  | 0.91 (0.65, 1.26) | 0.534 |
| Q3 | 1.13 (0.80, 1.58) | 0.478 |  | 1.08 (0.76, 1.55) | 0.654 |  | 1.10 (0.72, 1.67) | 0.637 |
| Q4 | 1.22 (0.91, 1.63) | 0.175 |  | 1.08 (0.77, 1.52) | 0.625 |  | 0.94 (0.68, 1.28) | 0.651 |
| *P* for trend |  | 0.167 |  |  | 0.489 |  |  | 0.983 |
| **DTA** |  |  |  |  |  |  |  |  |
| Q1 | *Ref* |  |  | *Ref* |  |  | *Ref* |  |
| Q2 | 1.18 (0.92, 1.52) | 0.178 |  | 1.32 (0.98, 1.77) | 0.064 |  | 1.03 (0.76, 1.39) | 0.846 |
| Q3 | 1.75 (1.35, 2.26) | <0.001 |  | 2.00 (1.46, 2.73) | <0.001 |  | 1.53 (1.07, 2.18) | 0.025 |
| Q4 | 1.25 (1.01, 1.55) | 0.038 |  | 1.35 (1.07, 1.72) | 0.015 |  | 0.93 (0.67, 1.29) | 0.618 |
| *P* for trend |  | 0.006 |  |  | 0.005 |  |  | 0.768 |
| **n-6 DPA** |  |  |  |  |  |  |  |  |
| Q1 | *Ref* |  |  | *Ref* |  |  | *Ref* |  |
| Q2 | 1.13 (0.77, 1.65) | 0.525 |  | 1.23 (0.84, 1.81) | 0.276 |  | 1.02 (0.65, 1.59) | 0.932 |
| Q3 | 1.32 (0.98, 1.79) | 0.066 |  | 1.39 (0.97, 2.01) | 0.073 |  | 1.13 (0.75, 1.71) | 0.502 |
| Q4 | 1.14 (0.79, 1.64) | 0.476 |  | 1.22 (0.82, 1.81) | 0.306 |  | 0.90 (0.56, 1.44) | 0.626 |
| *P* for trend |  | 0.291 |  |  | 0.212 |  |  | 0.738 |

**Note**: Model 1: Unadjusted model; Model 2: Adjusted for age, gender, race; Model 3: Adjusted for age, gender, race, smoking status, alcohol consumption, education BMI, diabetes, hypertension, and hyperlipidaemia. OR = Odds Ratio, CI = Confidence Interval.

**Table S4**: E-values ​​for the effects of omega-3, omega-6, their subtypes, and omega-6/omega-3 on periodontitis in the fully adjusted model.

| **E-Value for  OR Estimate** | **E-Value for Lower  limit of 95%CI** | **E-Value for Upper  limit of 95%CI** | **Variable** | **Level** | **Group** | **OR (95% CI)** |
| --- | --- | --- | --- | --- | --- | --- |
| 1.58 | NA | 1.00 | omega-3 | Q2 vs Q1 | Periodontitis vs Non-periodontitis | 0.75 (0.51, 1.10) |
| 1.74 | NA | 1.00 | omega-3 | Q3 vs Q1 | Periodontitis vs Non-periodontitis | 0.67 (0.45, 1.00) |
| 1.93 | NA | 1.39 | omega-3 | Q4 vs Q1 | Periodontitis vs Non-periodontitis | 0.59 (0.41, 0.85) |
| 1.36 | 1.00 | NA | omega-6 | Q2 vs Q1 | Periodontitis vs Non-periodontitis | 1.16 (0.82, 1.63) |
| 1.67 | 1.11 | NA | omega-6 | Q3 vs Q1 | Periodontitis vs Non-periodontitis | 1.45 (1.02, 2.07) |
| 1.27 | 1.00 | NA | omega-6 | Q4 vs Q1 | Periodontitis vs Non-periodontitis | 1.10 (0.78, 1.55) |
| 1.31 | 1.00 | NA | omega-6/omega-3 | Q2 vs Q1 | Periodontitis vs Non-periodontitis | 1.12 (0.75, 1.67) |
| 1.49 | 1.00 | NA | omega-6/omega-3 | Q3 vs Q1 | Periodontitis vs Non-periodontitis | 1.26 (0.91, 1.74) |
| 2.08 | 1.62 | NA | omega-6/omega-3 | Q4 vs Q1 | Periodontitis vs Non-periodontitis | 1.88 (1.37, 2.56) |
| 1.29 | 1.00 | NA | EPA | Q2 vs Q1 | Periodontitis vs Non-periodontitis | 1.11 (0.83, 1.50) |
| 1.27 | 1.00 | NA | EPA | Q3 vs Q1 | Periodontitis vs Non-periodontitis | 1.10 (0.82, 1.49) |
| 1.23 | NA | 1.00 | EPA | Q4 vs Q1 | Periodontitis vs Non-periodontitis | 0.93 (0.63, 1.38) |
| 1.11 | NA | 1.00 | ALN | Q2 vs Q1 | Periodontitis vs Non-periodontitis | 0.98 (0.64, 1.49) |
| 1.11 | NA | 1.00 | ALN | Q3 vs Q1 | Periodontitis vs Non-periodontitis | 0.98 (0.66, 1.47) |
| 1.25 | NA | 1.00 | ALN | Q4 vs Q1 | Periodontitis vs Non-periodontitis | 0.92 (0.59, 1.44) |
| 1.22 | 1.00 | NA | n-3 DPA | Q2 vs Q1 | Periodontitis vs Non-periodontitis | 1.07 (0.75, 1.52) |
| 1.27 | NA | 1.00 | n-3 DPA | Q3 vs Q1 | Periodontitis vs Non-periodontitis | 0.91 (0.65, 1.27) |
| 1.56 | NA | 1.00 | n-3 DPA | Q4 vs Q1 | Periodontitis vs Non-periodontitis | 0.76 (0.53, 1.07) |
| 1.90 | NA | 1.19 | DHA | Q2 vs Q1 | Periodontitis vs Non-periodontitis | 0.60 (0.37, 0.95) |
| 2.03 | NA | 1.33 | DHA | Q3 vs Q1 | Periodontitis vs Non-periodontitis | 0.55 (0.34, 0.88) |
| 2.28 | NA | 1.68 | DHA | Q4 vs Q1 | Periodontitis vs Non-periodontitis | 0.47 (0.32, 0.70) |
| 1.39 | 1.00 | NA | LNA | Q2 vs Q1 | Periodontitis vs Non-periodontitis | 1.18 (0.79, 1.76) |
| 1.65 | 1.00 | NA | LNA | Q3 vs Q1 | Periodontitis vs Non-periodontitis | 1.40 (0.94, 2.08) |
| 1.42 | 1.00 | NA | LNA | Q4 vs Q1 | Periodontitis vs Non-periodontitis | 1.20 (0.81, 1.80) |
| 1.29 | NA | 1.00 | GLA | Q2 vs Q1 | Periodontitis vs Non-periodontitis | 0.90 (0.65, 1.24) |
| 1.31 | 1.00 | NA | GLA | Q3 vs Q1 | Periodontitis vs Non-periodontitis | 1.12 (0.80, 1.57) |
| 1.18 | 1.00 | NA | GLA | Q4 vs Q1 | Periodontitis vs Non-periodontitis | 1.05 (0.65, 1.71) |
| 1.81 | 1.18 | NA | EDA | Q2 vs Q1 | Periodontitis vs Non-periodontitis | 1.56 (1.05, 2.31) |
| 1.52 | 1.00 | NA | EDA | Q3 vs Q1 | Periodontitis vs Non-periodontitis | 1.29(0.90, 1.84) |
| 1.34 | 1.00 | NA | EDA | Q4 vs Q1 | Periodontitis vs Non-periodontitis | 1.14 (0.76, 1.71) |
| 1.21 | NA | 1.00 | HGL | Q2 vs Q1 | Periodontitis vs Non-periodontitis | 0.94 (0.70, 1.26) |
| 1.32 | 1.00 | NA | HGL | Q3 vs Q1 | Periodontitis vs Non-periodontitis | 1.13 (0.78, 1.65) |
| 1.14 | 1.00 | NA | HGL | Q4 vs Q1 | Periodontitis vs Non-periodontitis | 1.03 (0.72, 1.45) |
| 1.27 | NA | 1.00 | AA | Q2 vs Q1 | Periodontitis vs Non-periodontitis | 0.91 (0.65, 1.26) |
| 1.28 | 1.00 | NA | AA | Q3 vs Q1 | Periodontitis vs Non-periodontitis | 1.10 (0.72, 1.67) |
| 1.21 | NA | 1.00 | AA | Q4 vs Q1 | Periodontitis vs Non-periodontitis | 0.94 (0.68, 1.28) |
| 1.14 | 1.00 | NA | DTA | Q2 vs Q1 | Periodontitis vs Non-periodontitis | 1.03 (0.76, 1.39) |
| 1.78 | 1.22 | NA | DTA | Q3 vs Q1 | Periodontitis vs Non-periodontitis | 1.53 (1.07, 2.18) |
| 1.23 | NA | 1.00 | DTA | Q4 vs Q1 | Periodontitis vs Non-periodontitis | 0.93 (0.67, 1.29) |
| 1.11 | 1.00 | NA | n-6 DPA | Q2 vs Q1 | Periodontitis vs Non-periodontitis | 1.02 (0.65, 1.59) |
| 1.32 | 1.00 | NA | n-6 DPA | Q3 vs Q1 | Periodontitis vs Non-periodontitis | 1.13 (0.75, 1.71) |
| 1.29 | NA | 1.00 | n-6 DPA | Q4 vs Q1 | Periodontitis vs Non-periodontitis | 0.90 (0.56, 1.44) |

**Table S5**: Characteristics of participants in NHANES 2011-2014 according to periodontitis

status after PSM.

| **Characteristic** | **Overall (N = 1560)** | **Non-periodontitis group  (N = 780)** | **Periodontitis group  (N = 780)** | ***P*** |
| --- | --- | --- | --- | --- |
| **Age (years)** | 52.00 (42.00, 63.00) | 53.00 (41.00, 63.00) | 52.00 (43.00, 63.00) | 0.940 |
| **Gender** |  |  |  | 0.683 |
| Female | 773 (45.67) | 391 (45.00) | 382 (46.38) |  |
| Male | 787 (54.33) | 389 (55.00) | 398 (53.62) |  |
| **Race** |  |  |  | 0.759 |
| Non-Hispanic White | 315 (11.63) | 154 (11.33) | 161 (11.95) |  |
| Other races | 630 (65.81) | 312 (66.26) | 318 (65.33) |  |
| Non-Hispanic Black | 157 (5.75) | 76 (5.27) | 81 (6.26) |  |
| Other Hispanic | 458 (16.81) | 238 (17.14) | 220 (16.46) |  |
| **Education** |  |  |  | 0.694 |
| Less than high school | 326 (17.21) | 157 (15.20) | 169 (19.36) |  |
| High school | 355 (24.24) | 174 (25.27) | 181 (23.13) |  |
| More than high school | 879 (58.55) | 449 (59.53) | 430 (57.50) |  |
| **BMI** |  |  |  | 0.905 |
| Underweight/normal | 880 (51.67) | 445 (51.81) | 435 (51.51) |  |
| Overweight | 406 (29.53) | 201 (29.31) | 205 (29.77) |  |
| Obese | 274 (18.80) | 134 (18.88) | 140 (18.72) |  |
| **Smoking status** |  |  |  | 0.982 |
| Never | 239 (11.43) | 115 (11.30) | 124 (11.57) |  |
| Former | 340 (19.16) | 172 (18.76) | 168 (19.59) |  |
| Now | 480 (34.17) | 243 (35.99) | 237 (32.21) |  |
| **Alcohol** **consumption** | 216 (15.51) | 110 (15.10) | 106 (15.94) |  |
| Never | 285 (19.73) | 140 (18.85) | 145 (20.68) |  |
| Former |  |  |  | 0.918 |
| Mild | 416 (23.91) | 209 (23.83) | 207 (23.99) |  |
| Moderate | 516 (33.84) | 262 (34.89) | 254 (32.72) |  |
| Heavy | 628 (42.25) | 309 (41.29) | 319 (43.29) |  |
| **Diabetes** |  |  |  | 0.417 |
| No | 346 (19.20) | 164 (16.93) | 182 (21.63) |  |
| Yes | 1214 (80.80) | 616 (83.07) | 598 (78.37) |  |
| **Hypertension** |  |  |  | 0.807 |
| No | 832 (53.79) | 420 (53.73) | 412 (53.85) |  |
| Yes | 728 (46.21) | 360 (46.27) | 368 (46.15) |  |
| **Hyperlipidaemia** |  |  |  | 0.789 |
| No | 376 (22.90) | 185 (21.59) | 191 (24.30) |  |
| Yes | 1184 (77.10) | 595 (78.41) | 589 (75.70) |  |

**Table S6**: Weighted logistic regression analysis of the association between PUFA and periodontitis after PSM.

| **Characteristic** | **Model 1** | |  | **Model 2** | |  | **Model 3** | |
| --- | --- | --- | --- | --- | --- | --- | --- | --- |
|  | **OR (95% CI)** | ***P*** |  | **OR (95% CI)** | ***P*** |  | **OR (95% CI)** | ***P*** |
| **Omega-3** |  |  |  |  |  |  |  |  |
| Q1 | *Ref* |  |  | *Ref* |  |  | *Ref* |  |
| Q2 | 0.85 (0.52,1.38) | 0.492 |  | 0.83 (0.50,1.36) | 0.441 |  | 0.82 (0.48,1.40) | 0.431 |
| Q3 | 0.68 (0.43,1.09) | 0.104 |  | 0.66 (0.42,1.06) | 0.084 |  | 0.68 (0.42,1.09) | 0.102 |
| Q4 | 0.62 (0.40,0.96) | 0.031 |  | 0.60 (0.38,0.93) | 0.025 |  | 0.62 (0.39,1.00) | 0.050 |
| *P* for trend |  | 0.017 |  |  | 0.012 |  |  | 0.029 |
| **Omega-6** |  |  |  |  |  |  |  |  |
| Q1 | *Ref* |  |  | *Ref* |  |  | *Ref* |  |
| Q2 | 1.12 (0.76,1.63) | 0.555 |  | 1.11 (0.75,1.64) | 0.580 |  | 1.13 (0.75,1.72) | 0.522 |
| Q3 | 1.32 (0.88,2.00) | 0.173 |  | 1.32 (0.87,2.00) | 0.185 |  | 1.45 (0.95,2.21) | 0.082 |
| Q4 | 0.92 (0.67,1.27) | 0.614 |  | 0.92 (0.66,1.28) | 0.611 |  | 1.02 (0.74,1.39) | 0.911 |
| *P* for trend |  | 0.903 |  |  | 0.896 |  |  | 0.558 |
| **Omega-6/omega-3** |  |  |  |  |  |  |  |  |
| Q1 | *Ref* |  |  | *Ref* |  |  | *Ref* |  |
| Q2 | 1.27 (0.85,1.88) | 0.231 |  | 1.29 (0.86,1.92) | 0.210 |  | 1.24 (0.78,1.98) | 0.333 |
| Q3 | 1.25 (0.91,1.72) | 0.155 |  | 1.29 (0.93,1.79) | 0.116 |  | 1.24 (0.88,1.75) | 0.195 |
| Q4 | 1.77 (1.21,2.58) | 0.004 |  | 1.87 (1.29,2.72) | 0.002 |  | 1.79 (1.19,2.69) | 0.009 |
| *P* for trend |  | 0.006 |  |  | 0.002 |  |  | 0.006 |

**Note**: Model 1: Unadjusted model; Model 2: Adjusted for age, gender, race; Model 3: Adjusted for age, gender, race, smoking status, alcohol consumption, education, BMI, diabetes, hypertension, and hyperlipidaemia. OR = Odds Ratio, CI = Confidence Interval.

**Table S7**: Weighted logistic regression analysis of the associations between omega-3 subclass fatty acids and periodontitis after PSM.

| **Characteristic** | **Model 1** | |  | **Model 2** | |  | **Model 3** | |
| --- | --- | --- | --- | --- | --- | --- | --- | --- |
|  | **OR (95% CI)** | ***P*** |  | **OR (95% CI)** | ***P*** |  | **OR (95% CI)** | ***P*** |
| **EPA** |  |  |  |  |  |  |  |  |
| Q1 | *Ref* |  |  | *Ref* |  |  | *Ref* |  |
| Q2 | 0.92 (0.65,1.29) | 0.615 |  | 0.91 (0.65,1.28) | 0.580 |  | 0.93 (0.64,1.35) | 0.678 |
| Q3 | 0.93 (0.69,1.26) | 0.644 |  | 0.93 (0.68,1.26) | 0.607 |  | 0.98 (0.68,1.41) | 0.921 |
| Q4 | 0.84 (0.53,1.31) | 0.427 |  | 0.83 (0.52,1.33) | 0.422 |  | 0.89 (0.53,1.49) | 0.629 |
| *P* for trend |  | 0.428 |  |  | 0.428 |  |  | 0.679 |
| **ALN** |  |  |  |  |  |  |  |  |
| Q1 | *Ref* |  |  | *Ref* |  |  | *Ref* |  |
| Q2 | 0.99 (0.70,1.39) | 0.940 |  | 0.99 (0.70,1.40) | 0.955 |  | 1.00 (0.67,1.48) | 0.993 |
| Q3 | 0.98 (0.67,1.44) | 0.929 |  | 0.99 (0.67,1.47) | 0.948 |  | 1.02 (0.66,1.57) | 0.925 |
| Q4 | 0.91 (0.64,1.28) | 0.572 |  | 0.91 (0.64,1.32) | 0.618 |  | 0.93 (0.61,1.43) | 0.731 |
| *P* for trend |  | 0.605 |  |  | 0.65 |  |  | 0.773 |
| **n-3 DPA** |  |  |  |  |  |  |  |  |
| Q1 | *Ref* |  |  | *Ref* |  |  | *Ref* |  |
| Q2 | 0.87 (0.58,1.31) | 0.496 |  | 0.86 (0.58,1.29) | 0.463 |  | 0.88 (0.58,1.35) | 0.529 |
| Q3 | 0.89 (0.65,1.24) | 0.485 |  | 0.89 (0.64,1.23) | 0.459 |  | 0.91 (0.64,1.30) | 0.583 |
| Q4 | 0.65 (0.48,0.89) | 0.009 |  | 0.65 (0.47,0.89) | 0.010 |  | 0.67 (0.47,0.96) | 0.031 |
| *P* for trend |  | 0.009 |  |  | 0.012 |  |  | 0.032 |
| **DHA** |  |  |  |  |  |  |  |  |
| Q1 | *Ref* |  |  | *Ref* |  |  | *Ref* |  |
| Q2 | 0.62 (0.34,1.12) | 0.107 |  | 0.60 (0.33,1.10) | 0.093 |  | 0.61 (0.33,1.12) | 0.099 |
| Q3 | 0.57 (0.33,0.99) | 0.046 |  | 0.54 (0.31,0.93) | 0.028 |  | 0.55 (0.32,0.95) | 0.035 |
| Q4 | 0.50 (0.31,0.79) | 0.004 |  | 0.46 (0.29,0.73) | 0.002 |  | 0.48 (0.30,0.77) | 0.005 |
| *P* for trend |  | 0.005 |  |  | 0.002 |  |  | 0.005 |

**Note**: Model 1: Unadjusted model; Model 2: Adjusted for age, gender, race; Model 3: Adjusted for age, gender, race, smoking status, alcohol consumption, education, BMI, diabetes, hypertension, and hyperlipidaemia. OR = Odds Ratio, CI = Confidence Interval.

**Table S8**: Weighted logistic regression analysis of the associations between omega-6 subclass fatty acids and periodontitis after PSM.

| **Characteristic** | **Model 1** | |  | **Model 2** | |  | **Model 3** | |
| --- | --- | --- | --- | --- | --- | --- | --- | --- |
|  | **OR (95% CI)** | ***P*** |  | **OR (95% CI)** | ***P*** |  | **OR (95% CI)** | ***P*** |
| **LNA** |  |  |  |  |  |  |  |  |
| Q1 | *Ref* |  |  | *Ref* |  |  | *Ref* |  |
| Q2 | 1.13 (0.79,1.63) | 0.487 |  | 1.14 (0.78,1.64) | 0.486 |  | 1.17 (0.79,1.73) | 0.388 |
| Q3 | 1.19 (0.79,1.78) | 0.397 |  | 1.18 (0.78,1.79) | 0.408 |  | 1.26 (0.83,1.93) | 0.254 |
| Q4 | 0.91 (0.65,1.29) | 0.602 |  | 0.92 (0.64,1.31) | 0.616 |  | 0.99 (0.70,1.42) | 0.961 |
| *P* for trend |  | 0.688 |  |  | 0.701 |  |  | 0.872 |
| **GLA** |  |  |  |  |  |  |  |  |
| Q1 | *Ref* |  |  | *Ref* |  |  | *Ref* |  |
| Q2 | 0.98 (0.65,1.45) | 0.899 |  | 0.97 (0.65,1.45) | 0.890 |  | 0.99 (0.66,1.49) | 0.962 |
| Q3 | 1.11 (0.79,1.55) | 0.535 |  | 1.11 (0.79,1.56) | 0.541 |  | 1.14 (0.78,1.68) | 0.466 |
| Q4 | 0.99 (0.66,1.49) | 0.957 |  | 0.99 (0.65,1.50) | 0.961 |  | 1.04 (0.64,1.69) | 0.847 |
| *P* for trend |  | 0.874 |  |  | 0.87 |  |  | 0.675 |
| **EDA** |  |  |  |  |  |  |  |  |
| Q1 | *Ref* |  |  | *Ref* |  |  | *Ref* |  |
| Q2 | 1.15 (0.77,1.72) | 0.485 |  | 1.15 (0.77,1.71) | 0.485 |  | 1.13 (0.73,1.74) | 0.556 |
| Q3 | 0.98 (0.68,1.40) | 0.890 |  | 0.97 (0.68,1.39) | 0.880 |  | 0.99 (0.66,1.48) | 0.957 |
| Q4 | 0.94 (0.69,1.30) | 0.706 |  | 0.94 (0.69,1.29) | 0.705 |  | 0.96 (0.67,1.37) | 0.789 |
| *P* for trend |  | 0.474 |  |  | 0.478 |  |  | 0.615 |
| **HGL** |  |  |  |  |  |  |  |  |
| Q1 | *Ref* |  |  | *Ref* |  |  | *Ref* |  |
| Q2 | 0.87 (0.59,1.29) | 0.482 |  | 0.87 (0.58,1.30) | 0.473 |  | 0.89 (0.58,1.37) | 0.566 |
| Q3 | 1.07 (0.78,1.47) | 0.666 |  | 1.07 (0.77,1.50) | 0.675 |  | 1.13 (0.78,1.62) | 0.490 |
| Q4 | 0.99 (0.78,1.26) | 0.945 |  | 0.99 (0.77,1.28) | 0.948 |  | 1.04 (0.76,1.43) | 0.792 |
| *P* for trend |  | 0.668 |  |  | 0.69 |  |  | 0.499 |
| **AA** |  |  |  |  |  |  |  |  |
| Q1 | *Ref* |  |  | *Ref* |  |  | *Ref* |  |
| Q2 | 1.06 (0.74,1.53) | 0.740 |  | 1.06 (0.72,1.55) | 0.767 |  | 1.07 (0.72,1.59) | 0.709 |
| Q3 | 1.10 (0.75,1.59) | 0.624 |  | 1.08 (0.73,1.61) | 0.677 |  | 1.14 (0.74,1.74) | 0.515 |
| Q4 | 1.00 (0.69,1.46) | 0.996 |  | 0.99 (0.67,1.45) | 0.948 |  | 1.04 (0.71,1.52) | 0.841 |
| *P* for trend |  | 0.953 |  |  | 0.985 |  |  | 0.781 |
| **DTA** |  |  |  |  |  |  |  |  |
| Q1 | *Ref* |  |  | *Ref* |  |  | *Ref* |  |
| Q2 | 1.20 (0.87,1.66) | 0.259 |  | 1.21 (0.87,1.68) | 0.255 |  | 1.17 (0.83,1.65) | 0.326 |
| Q3 | 1.51 (1.05,2.15) | 0.026 |  | 1.51 (1.04,2.18) | 0.031 |  | 1.53 (1.01,2.31) | 0.045 |
| Q4 | 0.94 (0.70,1.27) | 0.691 |  | 0.95 (0.71,1.28) | 0.733 |  | 0.95 (0.67,1.34) | 0.735 |
| *P* for trend |  | 0.907 |  |  | 0.905 |  |  | 0.887 |
| **n-6 DPA** |  |  |  |  |  |  |  |  |
| Q1 | *Ref* |  |  | *Ref* |  |  | *Ref* |  |
| Q2 | 0.90 (0.59,1.38) | 0.627 |  | 0.90 (0.58,1.39) | 0.616 |  | 0.89 (0.56,1.40) | 0.575 |
| Q3 | 1.07 (0.72,1.57) | 0.742 |  | 1.06 (0.70,1.59) | 0.788 |  | 1.05 (0.70,1.59) | 0.790 |
| Q4 | 0.98 (0.64,1.50) | 0.935 |  | 0.97 (0.63,1.50) | 0.902 |  | 0.97 (0.63,1.49) | 0.876 |
| *P* for trend |  | 0.866 |  |  | 0.903 |  |  | 0.911 |

**Note**: Model 1: Unadjusted model; Model 2: Adjusted for age, gender, race; Model 3: Adjusted for age, gender, race, smoking status, alcohol consumption, education, BMI, diabetes, hypertension, and hyperlipidaemia. OR = Odds Ratio, CI = Confidence Interval.

**Table S9**: The heterogeneity, pleiotropy test of PUFA on periodontitis.

| **Exposure** | **Outcome** | **Method** | **Nsnp** | **Heterogeneity** | **Pleiotropy_test** | **F_low** | **F_high** |
| --- | --- | --- | --- | --- | --- | --- | --- |
|  |  |  |  | **Q_pval** | **pval** |  |  |
| omega-3 | Chronic periodontitis | IVW | 48 | 0.006 | 0.434 | 26.163 | 6315.263 |
|  |  | MR Egger | 48 | 0.006 |  |  |  |
|  |  | Weighted median | 48 | NA |  |  |  |
| omega-6/omega-3 | Chronic periodontitis | IVW | 34 | 0.068 | 0.684 | 28.311 | 7582.712 |
|  |  | MR Egger | 34 | 0.056 |  |  |  |
|  |  | Weighted median | 34 | NA |  |  |  |
| omega-6 | Chronic periodontitis | IVW | 52 | 0.443 | 0.321 | 26.909 | 713.157 |
|  |  | MR Egger | 52 | 0.443 |  |  |  |
|  |  | Weighted median | 52 | NA |  |  |  |

**Table S10**: The heterogeneity, pleiotropy test of periodontitis on PUFA.

| **Exposure** | **Outcome** | **Method** | **Nsnp** | **Heterogeneity** | **Pleiotropy_test** | **F_low** | **F_high** |
| --- | --- | --- | --- | --- | --- | --- | --- |
|  |  |  |  | **Q_pval** | **pval** |  |  |
| Chronic periodontitis | omega-3 | IVW | 24 | 0.473 | 0.494 | 19.572 | 24.722 |
|  |  | MR Egger | 24 |  |  |  |  |
|  |  | Weighted median | 24 |  |  |  |  |
| Chronic periodontitis | omega-6/omega-3 | IVW | 24 | 0.347 | 0.235 | 19.572 | 24.722 |
|  |  | MR Egger | 24 |  |  |  |  |
|  |  | Weighted median | 24 |  |  |  |  |
| Chronic periodontitis | omega-6 | IVW | 24 | 0.905 | 0.202 | 19.572 | 24.722 |
|  |  | MR Egger | 24 |  |  |  |  |
|  |  | Weighted median | 24 |  |  |  |  |
